# Exploiting the Synergy between Concentrated Polymer Brushes and Laser Surface Texturing to Achieve Durable Superlubricity

**DOI:** 10.1021/acsami.2c00725

**Published:** 2022-03-25

**Authors:** Sorin-Cristian Vlădescu, Chiharu Tadokoro, Mayu Miyazaki, Tom Reddyhoff, Takuo Nagamine, Ken Nakano, Shinya Sasaki, Yoshinobu Tsujii

**Affiliations:** † Tribology Group, Department of Mechanical Engineering, Imperial College London, South Kensington, Exhibition Road, London SW7 2AZ, U.K.; ‡ Department of Mechanical Engineering, Saitama University, 255 Shimo-Okubo, Sakura, Saitama 338-8570, Japan; § Faculty of Environment and Information Sciences, Yokohama National University, 79-7 Tokiwadai, Hodogaya, Yokohama 240-8501, Japan; ∥ Department of Mechanical Engineering, Tokyo University of Science, Tokyo 125-8585, Japan; ⊥ Institute for Chemical Research, Kyoto University, Gokasho, Uji, Kyoto 611-0011, Japan

**Keywords:** polymer brushes, laser surface texturing, sliding friction, film thickness, superlubricity

## Abstract

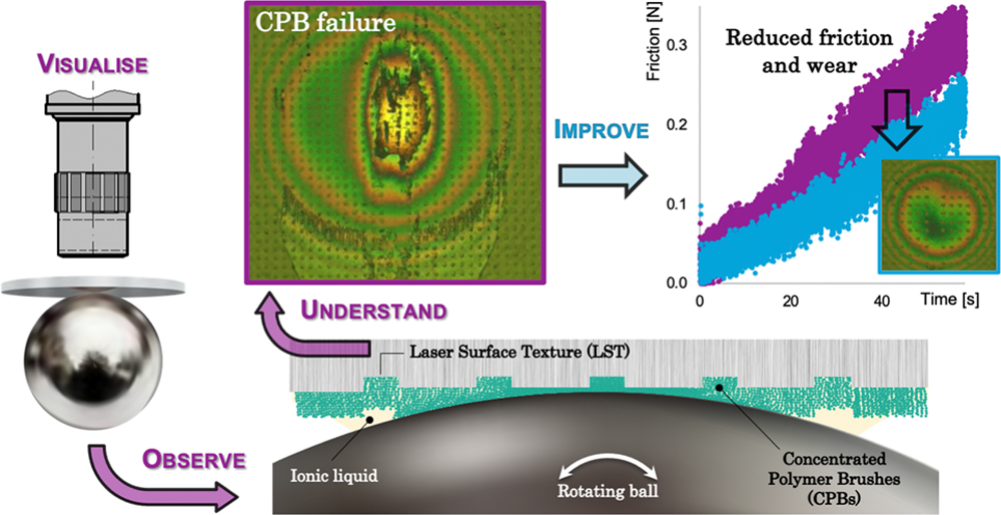

Friction continues to account for the bulk of energy losses in mechanical systems, with an estimated 23% of the world’s total energy consumption used to overcome friction. Concentrated polymer brushes (CPBs) have recently attracted significant scientific and industrial attention, given their ability to achieve superlubricity (i.e., coefficients of friction below 0.01); however, understanding the mechanistic interactions underlying their wear performance has been largely overlooked. Herein, we employ a custom-built optical test apparatus to investigate the inter-dependencies between CPBs and laser-produced surface texture (LST), assessing for the first time the friction, film thickness, and wear behavior in situ and simultaneously. Recent developments in picosecond laser etching allowed us to graft CPBs atop the finest laser-etched matrix of micron-sized dimples reported in literature to date. At low sliding speeds, combined CPB–LST reduces the coefficient of friction to 0.0006, while increasing the CPB durability by up to 34% through a lateral support mechanism offered by the textured micro-features. Furthermore, the imaging results shed light on CPB failure mechanisms. Both these mechanisms of lateral support and failure propagation impact the wear resistance of CPBs and are important in the development of CPBs for future applications (e.g., in low-speed bearings functioning under controlled abrasive wear conditions).

## 1 Introduction

Global energy demand is expected to see a 37% increase by 2040,^1^ while fossil fuel emissions are forecast to outweigh savings from renewables so that a catastrophic 2 °C rise in average temperature will be hard to avoid. In the quest to combat climate change, a high-impact measure is cutting the energy consumption of ∼1.2 billion vehicles in service today. A prime candidate is the estimated 57% of energy supplied to electric vehicles (EV) which is wasted on friction^2,3^ and extenuated by the exponential increase in the EV adoption rate. Also, in internal combustion engines (ICEs), friction losses still constitute 11.5% of the total fuel energy.^4^ Reducing mechanical friction through improved surfaces is thus one of the most effective ways to improve energy efficiency and reduce material waste. To this end, automobile and lubricant manufacturers have been implementing ways to reduce friction losses. These range from start–stop systems to limiting the ICE running time to more focused tribological approaches, such as the adoption of low-viscosity oils,^5^ polymeric e-lubricants (smart rheological),^6^ laser-produced surface texture coupled with mirror polishing of cylinder liners,^7^ and polymer-coated journal bearings.^8^

An effective way to facilitate sliding between components in contact is to densely anchor assemblies of polymer chains poly(methyl methacrylate) (PMMA) on the surface of solid materials. Recent advances in surface-initiated controlled radical polymerization allow the growth of uniform polymer brushes,^9−11^ while increasing the graft density by more than an order of magnitude compared to typical semi-dilute polymer brushes.^12^ This type of surface enhancement is referred to as concentrated polymer brushes (CPBs). Researchers from Kyoto University and Tsuruoka College have successfully shown that CPBs can reduce friction by 2 orders of magnitude, constructing a robust lubrication system, which can achieve superlubricity (μ_min_ < 10^–2^) in both nanoscopic^10−12^ and macroscopic tribological contacts.^13^ In addition, these CPBs enable a 10-fold increase in the thickness of the brush layer compared to conventional semi-diluted polymer brushes. This increase in thickness enhances the durability under sliding contacts^14^ and could thus be applied to mechanical components, including journal bearings, sealing devices, and piston assemblies.

Despite promising significant energy reductions, CPBs have escaped practical application because of two limitations: (i) lengthy exposure, high vacuum, and high temperature cause the rapid evaporation of most swelling agents such as organic solvents, leading to the loss of superlubricity and (ii) severe contact conditions reduce wear resistance capabilities. Research by Tsujii and Sato^15^ successfully addressed the former concern by employing an ionic liquid (IL) as a swelling agent. ILs help preserve the swollen state of polymer brushes over long periods of time and under high vacuum or high-temperature conditions due to the liquid’s minimal volatility and flammability, combined with relatively high thermal stability.^16^ Furthermore, researchers recently achieved a robust system by grafting IL polymer brushes onto smooth surfaces, which resulted in very low friction (μ_min_ ≈ 10^–3^) for applied normal loads as high as 15 N.^13^ The same order of magnitude of friction coefficient was recently recorded by Tadokoro et al.,^17^ who employed a custom-made apparatus to study the impact of well-swollen PMMA–CPBs on friction, clearance, and leakage rate in reciprocating seals.

Although recent polymer brush studies have shown exceptional frictional stability for up to 5000 cycles,^13,14^ limited preservation of brushes under severe high-pressure high-temperature operating conditions still prevents use on an industrial scale. To address this, the current study puts forward a combined friction and wear reducing surface modification technique, consisting of CPBs grafted onto surfaces initially laser-etched with a matrix of micron-sized features—the smallest texture dimensions so far reported in the tribology literature.

Applying laser surface texture (LST)—that is*,* features such as pockets or grooves—to the surface of components is a way of improving lubrication that has been investigated since the 1960s.^18^ The impact of this approach can be significant. In fact, it has been consistently shown to give friction reductions of up to 80% in controlled laboratory tests.^19−28^ Compared to other energy-saving solutions, LST is of low cost and easy to implement. It does not require components to be redesigned and can be incorporated into existing and new technologies. A recent series of studies at Imperial College has elucidated the tribological mechanisms associated with surface texture and explained earlier discrepancies, by highlighting the critical dependency on contact conditions. Under boundary and mixed lubrication conditions (i.e. when the lubricant layer between component surfaces is too thin to prevent solid–solid contact), pockets consistently boost fluid film thickness (probably as a result of cavitation-driven flow^29^ termed “inlet suction”) and thus drastically reduce friction.^30^ This is practically beneficial since many lubricated automotive components (pistons, cams, and gears, among others), routinely operate under mixed lubrication conditions. Specific pocket geometry criteria (shape, orientation, and spacing) have also been show to further reduce friction.^20,27^ When optimized in this way, surface texture coverage as low as 5% can generate friction reductions of up to 82%, compared to nontextured components.^21^ Alternatively, macroscale texture has recently been shown to reduce friction in the full-film regime, thanks to the shear area reduction mechanism.^31^ In addition, surface texture has been shown to reduce wear either by increased surface separation due to film thickness increase and/or by acting as debris traps.^20,21^

In a series of recent studies, Watanabe et al.^32,33^ combined IL swollen CPBs with micrometer-sized grooves and nano-periodic structures to achieve a significant increase in durability compared to CPBs grafted onto non-textured surfaces. It was shown that while grooves parallel to the direction of sliding act to increase the friction durability of CPBs by up to 36%,^32^ applying these micro-grooves on nano-periodic-structured surfaces improve the durability of CPBs by up to 90% compared with CPBs grafted onto non-textured surfaces.^33^

In a subsequent study, Miyazaki et al.^34^ examined the durability of PMMA–CPBs applied on substrates with chemically etched parallel grooves of different dimensions. Through a combination of lubricated sliding and nanoindentation tests, the authors proposed a durability enhancement mechanism created by the “layered structure” of the concentrated polymer brush. Nanoindentation tests showed that when grafted onto non-textured surfaces, CPBs display a structure comprising two layers: a diluted “surface layer” and a more concentrated “bulk layer”. When the CPBs were grafted onto a textured substrate, an additional, third layer (i.e., a “reinforced tough layer”) forms inside the concave space of the parallel grooves, acting to enhance the durability of the CPBs.

The aim of the current study was to improve friction and wear efficiency by understanding and exploring the inter-dependencies between CPBs and laser-produced surface textures. To achieve this, a novel test apparatus was designed and built at Saitama University to allow in situ visualization of CPB-collapsing behavior and shed light on previously hypothesized wear mechanisms of CPBs. Tests were conducted under the most extreme conditions that CPBs can endure (located on the static surface of a sliding contact pair and thus subject to constant high contact pressure throughout the test duration), with the aim of demonstrating their real-life potential to reduce energy losses and improve machine efficiency. This is underpinned by measurements to understand the mechanistic interactions between CPBs and LST and hence assess the potential of micron-size pockets to effectively reduce wear and consequently increase the life of CPBs by trapping debris particles (as shown by the authors recently for steel-on-steel reciprocating contacts^21^). Based on this, a new anti-wear strategy is put forward, in which femtosecond laser-textured micro-cavities suppress the propagation of CPB failure through the offered additional lateral support and thus increase the durability.

## 2 Description of the Test Apparatus

**Figure 1:**
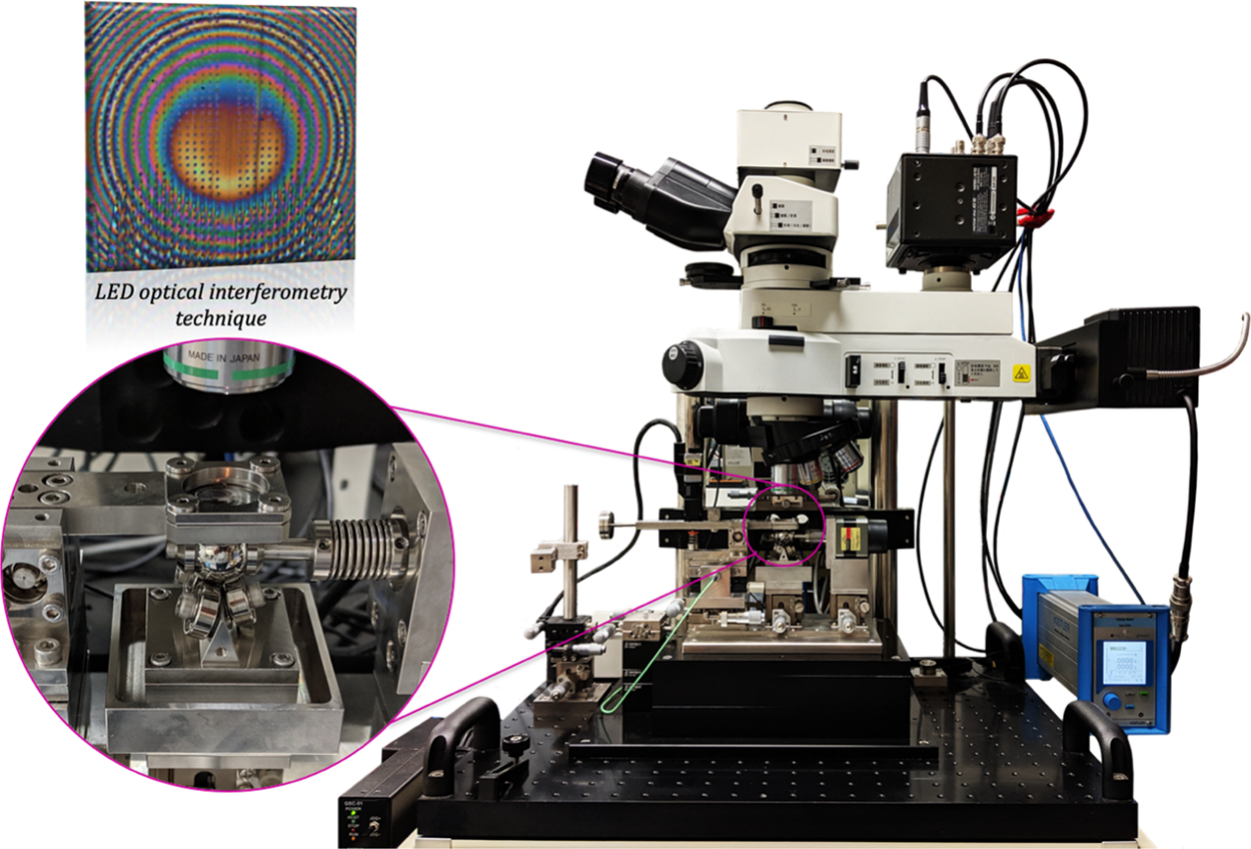
Layout of the custom-built test apparatus.

This study uses a custom-built test apparatus, designed, and built at Saitama University, to allow in situ and simultaneous measurement of key tribological parameters of a ball-on-disc contact (Figure 1). This test rig simulates high-pressure, elastohydrodynamic contact conditions as found in rolling bearings and gears and measures film thickness and wear (as described in ref (35)) of CPB-grafted, textured contacts. During each test, the ball specimen rolls on an arrangement of three ball bearings, driven by an electric motor through a flexible coupling. The counterpart specimen, a fused silica disc, is securely positioned inside a stationary holder, which also allows automatic normal loading via a counter-balancing mechanism facilitated by a loading sensor. As the ball specimen rolls against the static disc, the friction force is recorded using a high-sensitivity load cell, connected to a mechanism that holds the counter-balancing system.

To achieve nanometer film thickness measurements for various CPB–LST configurations, a custom version of the optical ultrathin-film interferometry technique was installed on the rig system. Before the radical polymerization process of the polymer brushes, the lower surface of the transparent fused silica disc was coated with a 20 nm-thick, semi-reflective chromium layer. This created the interference fringes observed in Figure 1 (detail—interferogram of a textured contact). As opposed to the classical ultrathin-film interferometry technique,^36,37^ no silica spacer layer was applied on top of the chromium layer, as this was replaced by the thickness of the CPBs. A combined LabVIEW-Scout computer program was used to determine the initial, static film thickness of the CPB–IL mixture as well as the mixture’s film thickness variation throughout the tests. Two methods were used to determine the CPB–IL mixture’s thickness: (i) a halogen light source that allows nanometer-size measurements of center film thickness (single point measurement, spot diameter: 20 μm) and (ii) a light-emitting diode (LED) three-chromatic system using red, blue, and green light sources to allow the three-dimensional mapping of the CPB–IL film thickness inside the contact. The former technique is the main tool used in the current study. However, short movie clips recorded by the LED system are shown in Section 4. Prior to each test session, a calibration procedure was carried out to carefully determine the dark and refractive spectra as well as the film thickness under static loading conditions.

To achieve an equal loading time for all disc samples and thus avoid differences in lubricant squeeze time and polymer brush compression, the contact was loaded for only 2 s prior to each test. A triggering system ensured that the high-speed camera reading from the spectrometer (for film thickness measurement) and isometric load cell (capturing frictional response) are accurately synchronized at the precise moment when the normal load is applied. A LabView program simultaneously captured real-time measurements of frictional force and film thickness.

## 3 Test Specimens and Experimental Procedure

**Figure 2:**
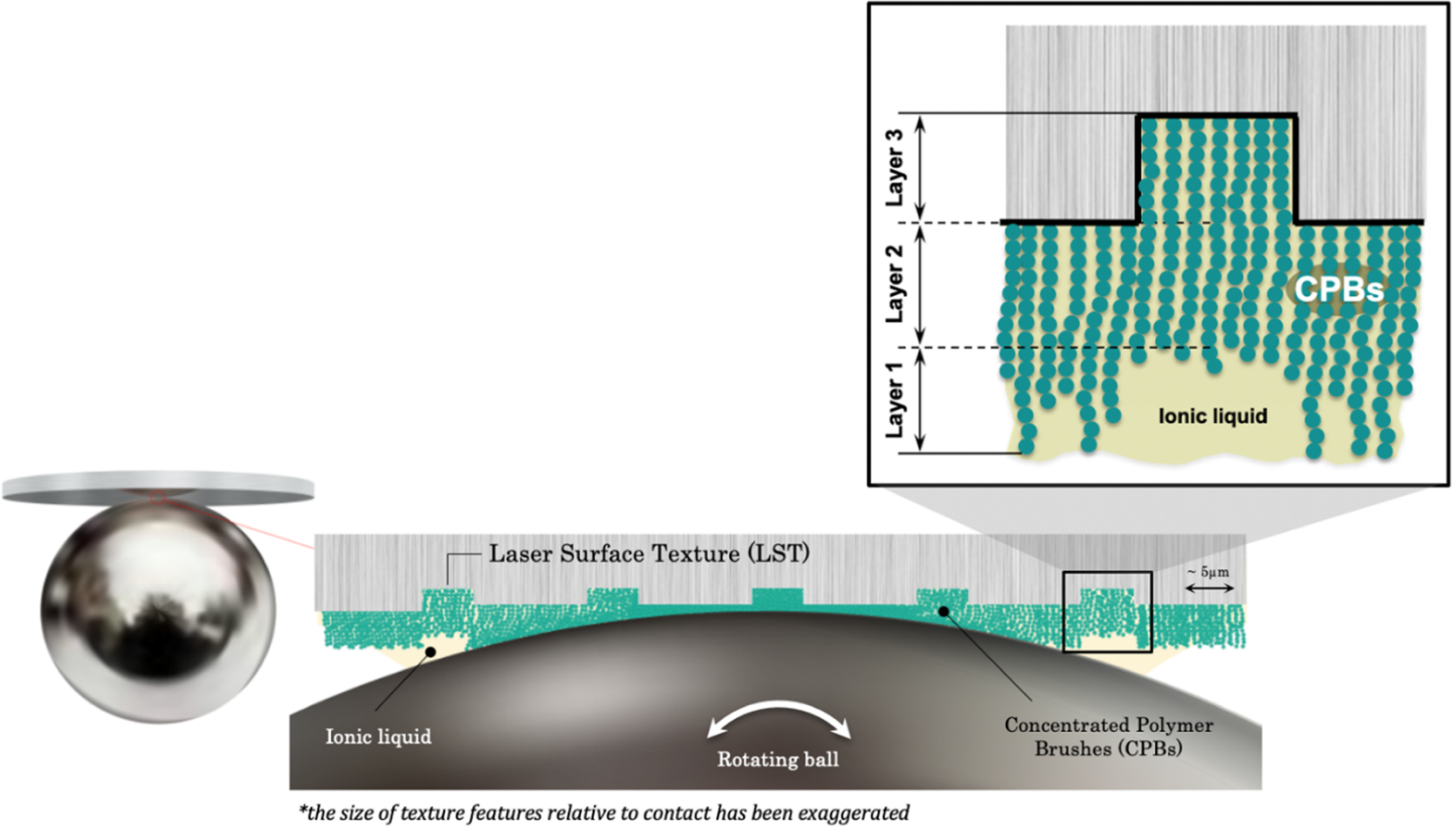
Schematic representation of the CPB-coated, LST ball-on-disc contact.

A schematic of the contact under investigation is shown in Figure 2. For each sliding test, a brand new, super-finished AISI 52100 (535A99) steel ball was used. The mirror-polished surface of the steel ball (19.05 mm in diameter) ensures that measurement stability is achieved both over time and between repeated tests. Excellent repeatability is shown later in this section.

The flat counter specimen, on which both LST and polymer brushes were applied, was a transparent fused silica disc of 25 mm in diameter and 2 mm thick (Figure 2). This high-purity fused silica (HPFS) Standard Grade material was used due to its high stiffness (Young’s modulus of 72.7 GPa) and excellent optical properties for interferometry imaging. The texturing was achieved using a femtosecond laser, designed and operated by Oxford Lasers Ltd, UK, while the surface-initiated controlled radical polymerization of the CPBs was executed at Kyoto University Institute for Chemical Research.

**Figure 3:**
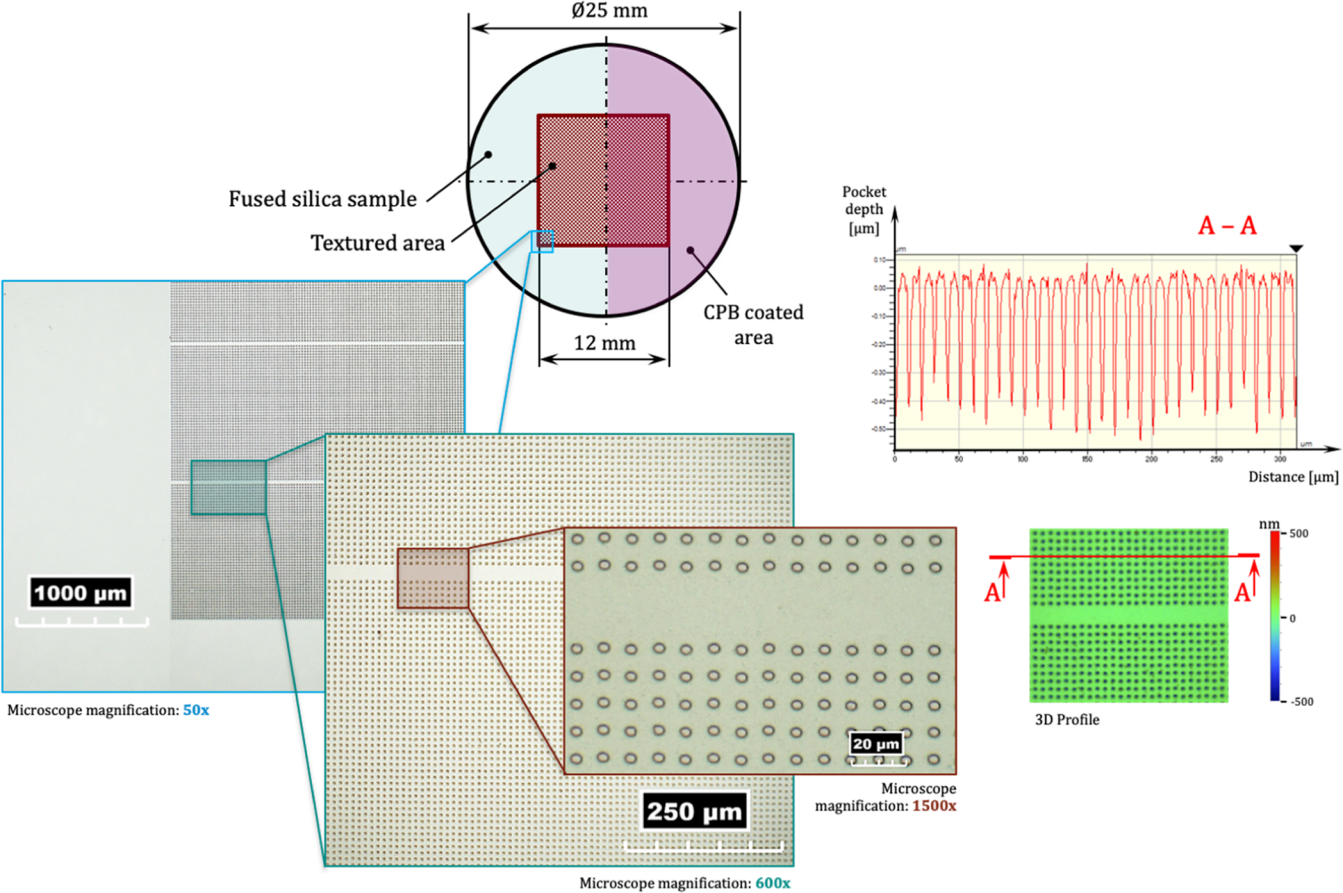
Two-dimensional and three-dimensional surface plots of the fused silica disc, showing the micro-pocket arrangement and the pocket-free zone, where film thickness is measured, the polymer grafted area, as well as the high-repeatability of the dimples’ geometrical features.

Laser texturing produced circular pockets covering a 12 × 12 mm area, located precisely at the center of the disc. Within this area, pocket-free strips that are 20 and 40 μm wide were located with a spacing of 1 mm (Figure 3). These zones are important as they allowed (i) accurate film thickness measurements along the center of the contact, avoiding pocket interference and (ii) wear behavior comparisons between textured and non-textured surfaces.

Considering the maximum height of the swollen polymer brushes (approximately 780 nm), two profile requirements of the textured features proved critical for correctly assessing the combined LST–CPB tribological behavior: (i) no pile up of melted material around the pocket edges and (ii) the laser-textured pockets have the exact required depth. Laser texturing meeting these criteria was produced for three of the four specimens (Figure 3, detail A–A shows an example with a radius of 5 μm and a pocket depth of 0.4 μm) recorded using a Hirox RH-2000 optical microscope as well as a two-dimensional section obtained using a Veeco Wyko NT9100 white light interferometer, which depicts pocket depth regularity. An exception involving material pileup around feature edges was produced on the sample with pocket radius 10 μm and depth 0.2 μm, the effects of which are investigated in Section 4. The femtosecond laser texturing process allowed the smallest pocket diameter so far recorded in the tribology literature to be achieved: more than 70 pockets were completely entrapped inside the 150 μm diameter contact at any given time.

CPBs of PMMA were grafted onto half of each fused silica disc surface, which allowed for a direct comparison between CPB-coated and non-coated surfaces, without the need to disassemble the sample and thus reducing any misalignment errors. The PMMA–CPBs were synthesized and characterized as detailed in^38,39^ and briefly described in the Supporting Information. The average film-thickness under dry condition, number-average molecular weight (*M*_n_), and dispersity (*D̵*) (as values of free polymers simultaneously produced in the CPB synthesis) of the studied CPBs were 257 nm, 7.5 × 105, and 1.23, respectively. The graft density (σ) and the surface occupancy (σ*) of the CPB were calculated to be 0.25 nm^–2^ and 0.14, respectively. An IL, *N*-(2-methoxyethyl)-*N*-methylpyrrolidinium bis(trifluoromethane sulfonyl)imide MEMP–TFSI, was used as a solvent for swelling the CPBs and as a lubricant. Prior to testing each CPB-coated surface, samples were kept covered in the IL MEMP–TFSI for a period of 24 h to allow uniform swelling of the brushes.

The geometrical parameters of the four textured configurations are presented in Table 1 (dimple depth and dimple radius, 1500× image). Each of these four LST specimens and a non-textured reference were tested both with and without CPB coatings, thus totaling ten LST–CPB configurations.

**Table 1:**
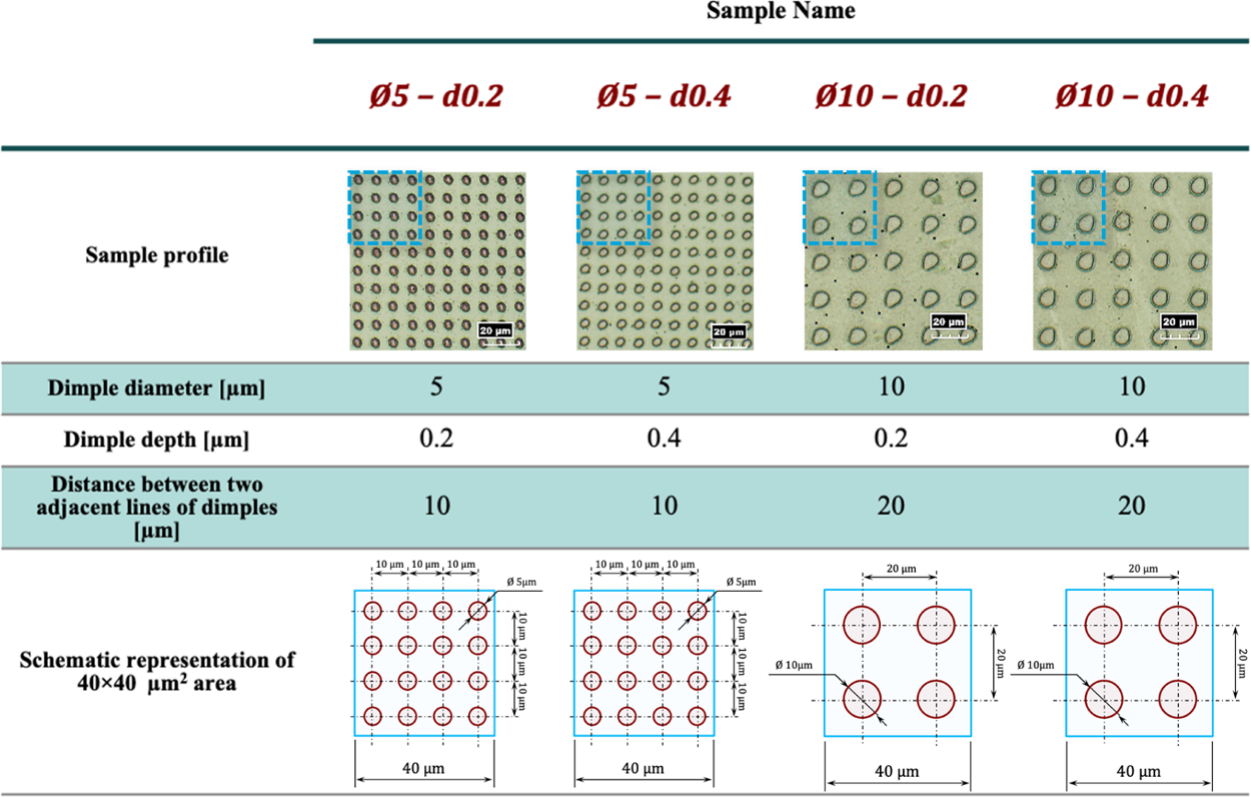
Geometric Parameters of the CPB–LST Combinations Tested

Since the ball specimen slides (with a surface speed of *u*_1_) against the stationary fused-silica disc (speed *u*_2_ = 0), in this test setup configuration, the lubricant entrainment speed [*U* = (*u*_1_ + *u*_2_)/2] always equals half of the surface speed of the ball. For clarity, this paper will only refer to the ball sliding speed (Δ*u* = *u*_1_ – *u*_2_), as this acts to determine the viscous friction. For each set of tests, the sliding speed was progressively varied between 0.2 and 2000 mm/s (i.e., a fresh specimen was used for each speed measurement), while keeping the applied normal load constant at 5 N. At the beginning of each test, a single 10 μL dose of IL was supplied to the contact using a micropipette.

## 4 Results and Discussion

This section demonstrates the benefits of CPBs in terms of sliding friction and their sliding distance/stress-related limitations. The impact of geometrical surface-textured parameters on friction force and film thickness are also characterized as well as the ability of micro-features to reduce CPB wear. Finally, results are summarized and a new friction and wear reduction mechanism for this combined CPB–LST is put forward.

### 4.1 Influence of CPBs on Friction Force and Their Wear Behavior

#### 4.1.1 Compression of the Diluted Surface Layer under Static Conditions

**Figure 4:**
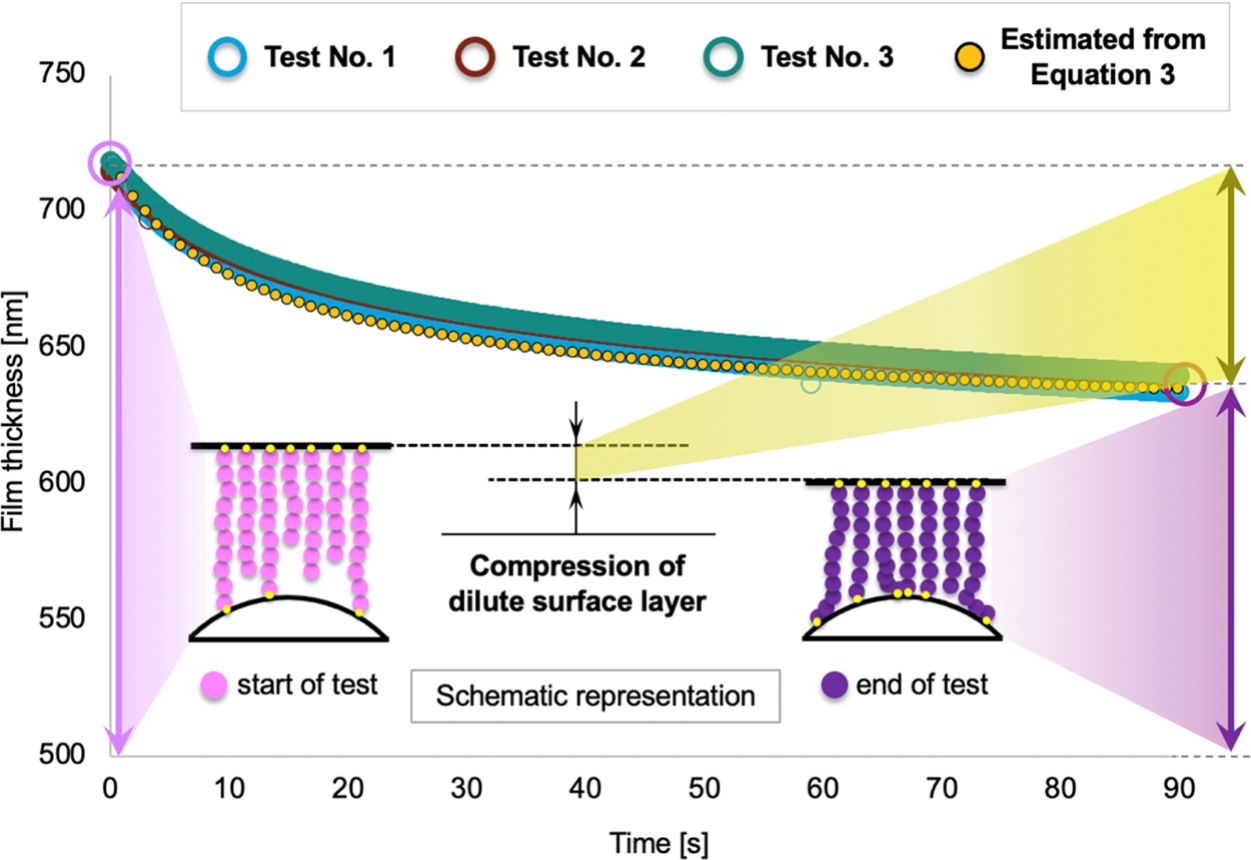
Film thickness decline over time for the CPB-coated, non-textured specimen; repeatability between three different tests. Inset—schematic representation of the time-dependent compression process of the diluted surface layer for a constant applied load of 5 N. The pictogram highlights the change between the initial position of the polymer brushes immediately after the contact is loaded and their final orientation at the end of the static test. At the start of the test, the load is supported by a small number of adhesion points and the surrounding IL; this increases gradually as the diluted surface layer is compressed and the load becomes carried by a more homogeneous, concentrated bulk layer. As the combined CPB-lubricant film decreases and the number of adhesion points grow, the diluted surface layer, crucial for the ultralow friction of CPBs,^33^ is gradually compressed to become a concentrated bulk layer. Red dotted line shows theoretical squeeze film thickness predicted by eq 3.

To investigate the time-dependent compression of the CPB “diluted surface layer” (i.e., a layer with a low elastic modulus initially identified by Miyazaki et al. in ref (34) following a series of nanoindentation tests), Figure 4 shows the combined CPB-lubricant film thickness variation recorded for three non-consecutive tests over a period of 90 s. These repeat tests were performed using a non-textured sample under static conditions (zero sliding speed) with an applied normal load of 5 N. In addition to the high measurement repeatability, this shows that the combined CPB-lubricant film (i.e., IL) gradually decreases over the 90 s period from a maximum swollen height of 715 nm to an average height of 637 nm. The gradual film reduction suggest that a squeeze flow process is more likely to be occurring than the rotation of the brushes.

This can be analyzed by considering a viscous fluid between plates, under zero sliding speed, which has a time-dependent film thickness predicted using Reynolds equation
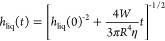
1where *h*_liq_(0) is the initial film thickness, *W* is the applied load, *R* is the radius of contact (estimated using Hertz theory to be ∼307 μm), and η is the viscosity. The film thickness, *h*_liq_, in eq 1 is that of the “liquid-like” dilute layer (consisting of polymer brushes in the IL solvent) as identified by Miyazaki et al. in ref (34) since it is the one that will flow out of the contact under load. Therefore, the total film thickness (which is measured and plotted in Figure 4) is given by
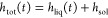
2where *h*_sol_ is the constant thickness of the “solid-like” concentrated bulk layer (which does not flow out of the contact). Combining eqs 1 and 2 gives
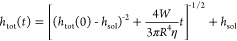
3

Equation 3 can then be fitted to the data in Figure 4 (as shown by the yellow dotted line), with the only adjustable constants being the viscosity, η, and the concentrated bulk layer thickness, *h*_sol_. This yields a viscosity value of ∼50 mPa s (which is similar to that of neat MEMP–TFSI^15^ and a concentrated brush layer thickness of ∼605 nm, which is consistent with independent indentation measurements^34^). The close match between eq 3 and the measured film thickness, combined with the reasonableness of the resulting brush thickness and viscosity estimates, supports the hypothesis that squeeze flow is responsible for the observed decay in film thickness. Note that the long duration of the process (occurring over 10 s of seconds) is a result of the CPB’s low stiffness and hence large contact radius, which requires time for the fluid to flow across.

#### 4.1.2 Impact of the Sliding Distance and Surface Texture on the Wear Behavior of CPBs

**Figure 5:**
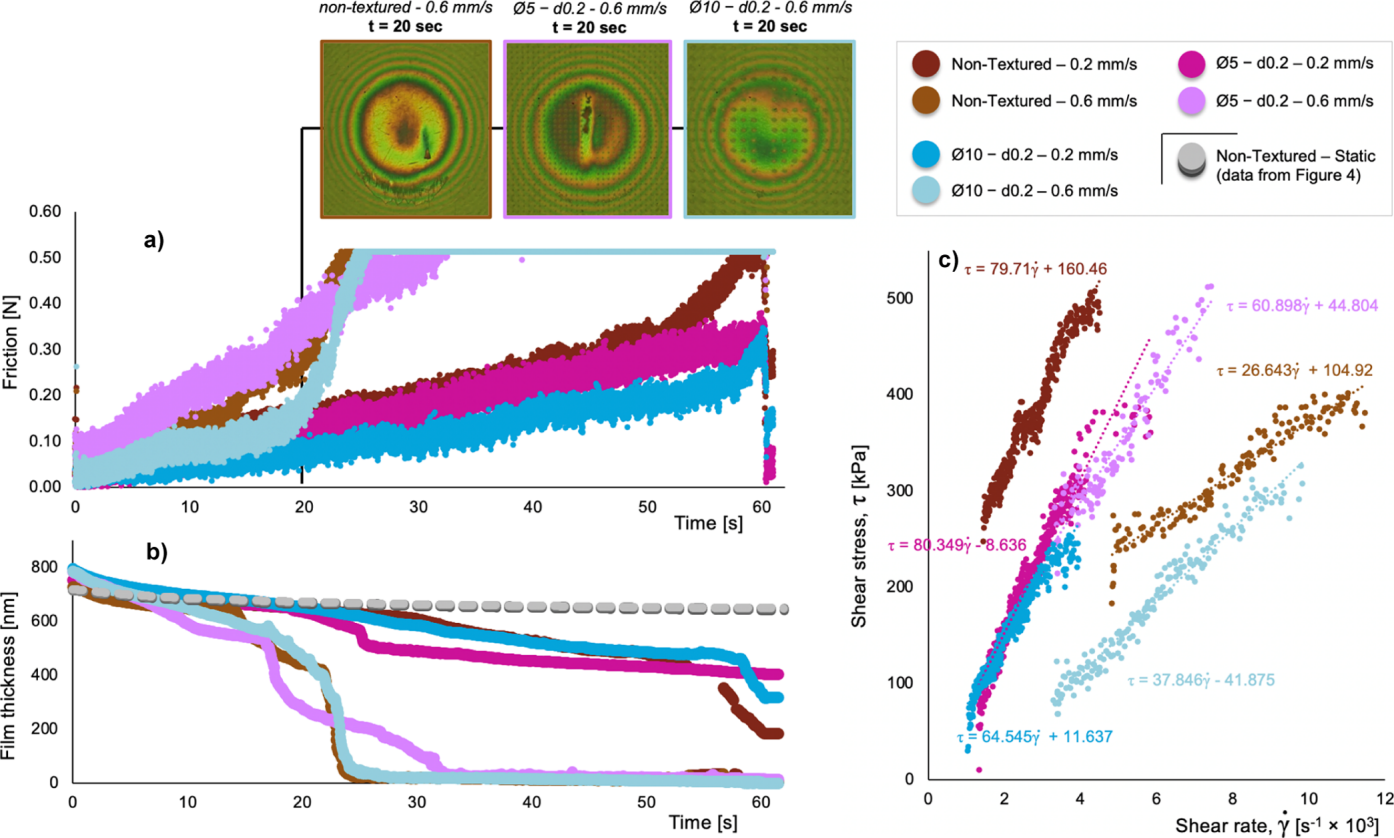
Simultaneous friction force (a) and film thickness (b) comparisons between all CPB-coated, textured and non-textured disc samples, recorded over the 60 s wear tests (test conditions: sliding speed: 0.2 and 0.6 mm/s; applied load: 5 N; lubricant temperature: 25 °C); Inset—interferometry images captured for all three samples after 20 s of sliding (sliding speed: 0.3 mm/s). (c) Shear stress versus strain rate; data replotted from Figure 5a,b.

To assess the wear behavior, a series of extended sliding tests (60 s each) was performed. The resulting frictional response of two textured patterns (Ø5 *– d*0.2 and Ø10 *– d*0.2) is compared against the non-textured, CPB-coated reference (Figure 5a). The combined CPB-IL film thickness is shown quantitatively (Figure 5b) as well as qualitatively (Figure 5a—details and Videos S2, S3, and S4). The textured patterns of identical depth and different diameter (5 and 10 μm) and also the smooth reference were tested at the two lowest speeds of 0.2 and 0.6 mm/s.

The film thickness from the static case (i.e.*,* data from Figure 4) is also shown in Figure 5b. Once the diluted surface layer of the CPBs is compressed/squeezed out and the combined CPB-lubricant is reduced to around ∼600 nm (i.e.*,* the thickness of the concentrated bulk layer), a close agreement is observed between the frictional response and the squeezing time of the more concentrated CPB layer (Figure 5).

Figure 5a shows that the Ø10 *– d*0.2 textured pattern displays both the lowest initial friction response and the greatest wear durability at both sliding speeds. The non-textured reference has the weakest performance and shows accelerated degradation after ∼50 s, with the film thickness gradually reducing to around 200 nm.

The three images attached to Figure 5a display the CPB film for all three samples, captured after 20 s of sliding at 0.6 mm/s. While for both non-textured and Ø5 *– d*0.2, the CPB collapse has already started; no sign of wear is apparent for the Ø10 *– d*0.2 textured pattern. However, as shown in Video S3, after 20 s, and as highlighted in Figure 5a, the CPB layer on the Ø10 *– d*0.2 sample collapses entirely in less than 4 s. Although the CPBs on the Ø5 *– d*0.2 sample are the first to show signs of degradation and the narrower micro-features delay the collapse, increasing the life of the polymer brushes by ∼10 s (5.5 mm). This behavior can also be observed in Figure 5b, which shows a prolonged resistance of the combined CPB-IL film thickness for the Ø5 *– d*0*.*2 sample when sliding at 0.6 mm/s.

For all textured surfaces, the collapse of the CPBs always commenced along the dimple-free zone at the center of the contact, where two consecutive lines of pockets were omitted during laser micromachining. We hypothesized in ref (34) that this wear reduction behavior due to surface texture, which can be seen in all corresponding videos, is generated by the presence of a third, “reinforced tough layer”, located inside the concave space of the textured micro-features, where the polymer chains are grafted onto both the bottom of the pockets and the “vertical” sidewall surfaces. This enhanced CPB durability that delays/decelerates wear is supported by the visual proof presented in Figure 5 and Videos S2–S4 (see whole videos in the Supporting Information).

Figure 5a,b shows that, for each sample, friction increases as film thickness (i.e.*,* shear rate) decreases. This may be due to the viscous nature of the film within the contact and can be investigated as follows. The friction data in Figure 5a can be converted into shear stress, τ, by dividing by the contact area (*A* = π*R*^2^ = π × (307 × 10^–6^)^2^ = 2.96 × 10^–7^ m^2^), and the measured film thickness  in Figure 5b can be converted to strain rate by γ̇ = Δ*u*/*h*_liq_ = Δ*u*/( – *h*_sol_), where *h*_sol_ is the concentrated brush layer thickness, which was estimated from Figure 4 to be ∼605 nm. The resulting plot of shear stress against the strain rate in Figure 5c shows a linear relationship between shear stress and strain rate suggesting Newtonian fluid behavior (τ = ηγ̇). There is variation between the slopes of these lines, which may be due to deviations in the polymer brush thickness (not always 605 nm as assumed). However, the average gradient (i.e.*,* a prediction of the viscosity) is 58 mPa s with a standard deviation of 20 mPa s. This is remarkably close to the 50 mPa s predicted by Figure 4 and confirmed by the literature^15^ and strongly suggests that the observed friction behavior is dominated by the viscous film behavior of the MEMP–TFSI solvent. It should also be noted, however, that the time until failure of the CPB-coated specimens in Figure 5a,b (denoted by an abrupt reduction in film thickness) is closely linked to the sliding distance rather than time, for both the 0.3 mm/s and the 0.6 mm/s sliding speeds tests; the total sliding distance to collapse was approximately 16 mm (i.e.*,* 2× the sliding speed took 1/2 the time to fail).

#### 4.1.3 Ability of CPBs to Reduce Friction and Possible Causes of Wear

Throughout this study, CPBs were tested under severe contact conditions, being at all times located on the stationary component of the tribo-pair. The same polymer brushes located inside the contact on initial loading were thus subjected to friction and wear throughout the entire test. Previous studies describing the lubrication mechanism of CPBs were performed under milder conditions, with polymer brushes generally located on the moving surface. In the latter situation, the contact is continuously replenished with fresh, fully swollen brushes. Moreover, the non-conformal point contact investigated in this study led to a high contact pressure of 25 MPa being consistently applied on the polymer brushes.

**Figure 6:**
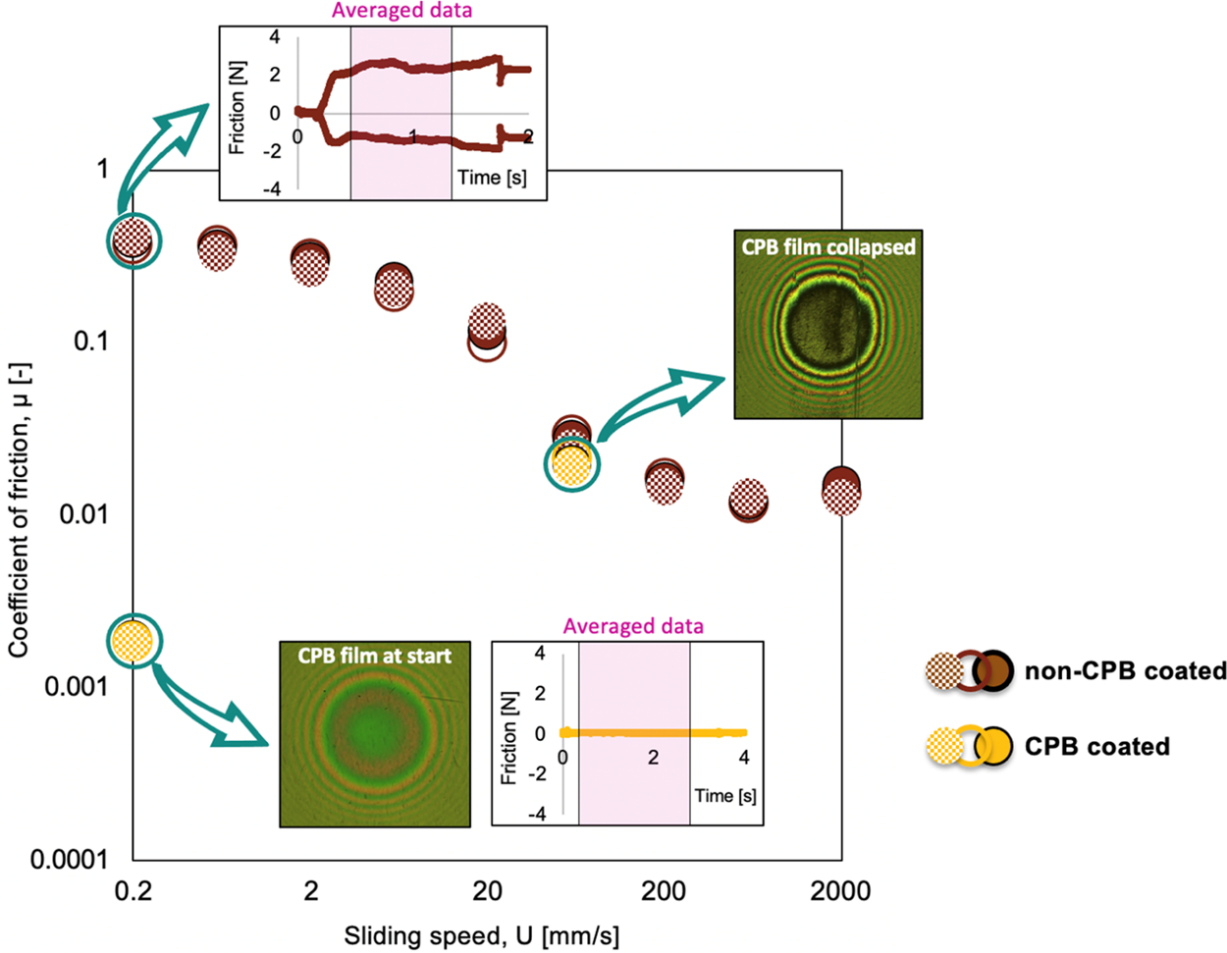
Stribeck curves showing the friction behavior for the non-CPB coated reference, plotted alongside the coefficient of friction recorded at 0.2 and 60 mm/s for the CPB-coated sample (picture insets showing interferometry images captured for the CPB-coated sample immediately after sliding movement begun and when CPB collapse. Graphical insets show friction force variation as recorded during the 0.2 mm/s step measurement—the red square representing the time interval where the friction was averaged. Applied load was kept constant at 5 N, while sliding speed was varied from 0.2 to 2000 mm/s.

It is not possible to plot standard Stribeck curves (friction vs speed) for the CPB-grafted samples in this study, since the coefficient of friction is not only a function of sliding speed but also of sliding distance and duration (as shown above). Hence, to assess the ability of CPBs to provide low friction, Figure 6 plots two sets of data, each obtained following three repetitions: (i) the frictional response recorded for the non-CPB coated, non-textured reference, obtained over a range of sliding speeds varying between 0.2 and 2000 mm/s (i.e.*,* classic Stribeck curves), and (ii) a limited frictional data set for the CPB coated, non-textured sample, consisting of the coefficient of friction during the initial step (sliding speed: 0.2 mm/s) and the frictional response at 60 mm/s, where the CPB film is instantly collapsed by the shear stress breaking the anchoring bonds between the brushes and the surface.

As expected, the non-CPB coated sample produces a standard Stribeck curve, showing the transitions between boundary, mixed, and hydrodynamic lubrication regimes. For the non-textured, non-CPB-coated sample, the highest friction is recorded at low speeds, as insufficient lubricant is entrained between the surfaces and load is supported by asperity contact. There is then a steep decrease in friction as speed increases, and more lubricant is entrained to separate the surfaces with an easily sheared IL layer (i.e.*,* mixed lubrication regime). Finally, as the sliding speed increases above 200 mm/s, the bearing shifts to the full film regime and friction rises again because of increased shearing of IL layers.

When comparing the CPB-coated sample with the non-coated reference, significant reductions in friction of up to 99.4% were observed during the initial sliding step (representing the difference in average friction of the data points recorded when the sliding speed was set at 0.2 mm/s, highlighted by the graphical insets shown in Figure 6). The low-speed friction of the CPB specimen is less than 0.01 and is sufficiently low to be classed as superlubricity.

Figure 6 also shows that the benefits from CPB grafting vanish at sliding speeds above 60 mm/s, which corresponds to the transition from mixed to a full film regime. To further understand the causes of this reduction in friction performance, the contact was viewed using the interferometry set-up. As shown in Figure 6 and Video S1, the CPBs were entirely removed from the surface in less than 1 s from the start of each test. It should be noted here that a fresh coated specimen was used for each measurement point.

After inspecting Figures 5 and 6, the following comments can be made about the failure of CPBs under submerged sliding conditions: (i) time to failure is proportional to the sliding distance (Figure 5) in agreement with an Archard coefficient type law, (ii) the IL is gradually squeezed out of the sliding contact (the higher the speed, the faster this happens), (iii) the shear stress (proportion to friction coefficient) increases as the film thickness decreases and may reach a level sufficient to cause scission of the brushes themselves or scission of the bonds between brushes and substrate, and (iv) localized failure occurs and leads to a stress concentration that causes other regions to follow a scraping type of wear.

#### 4.1.4 CPB Layered Structure Behavior with Increasing Sliding Speed

**Figure 7:**
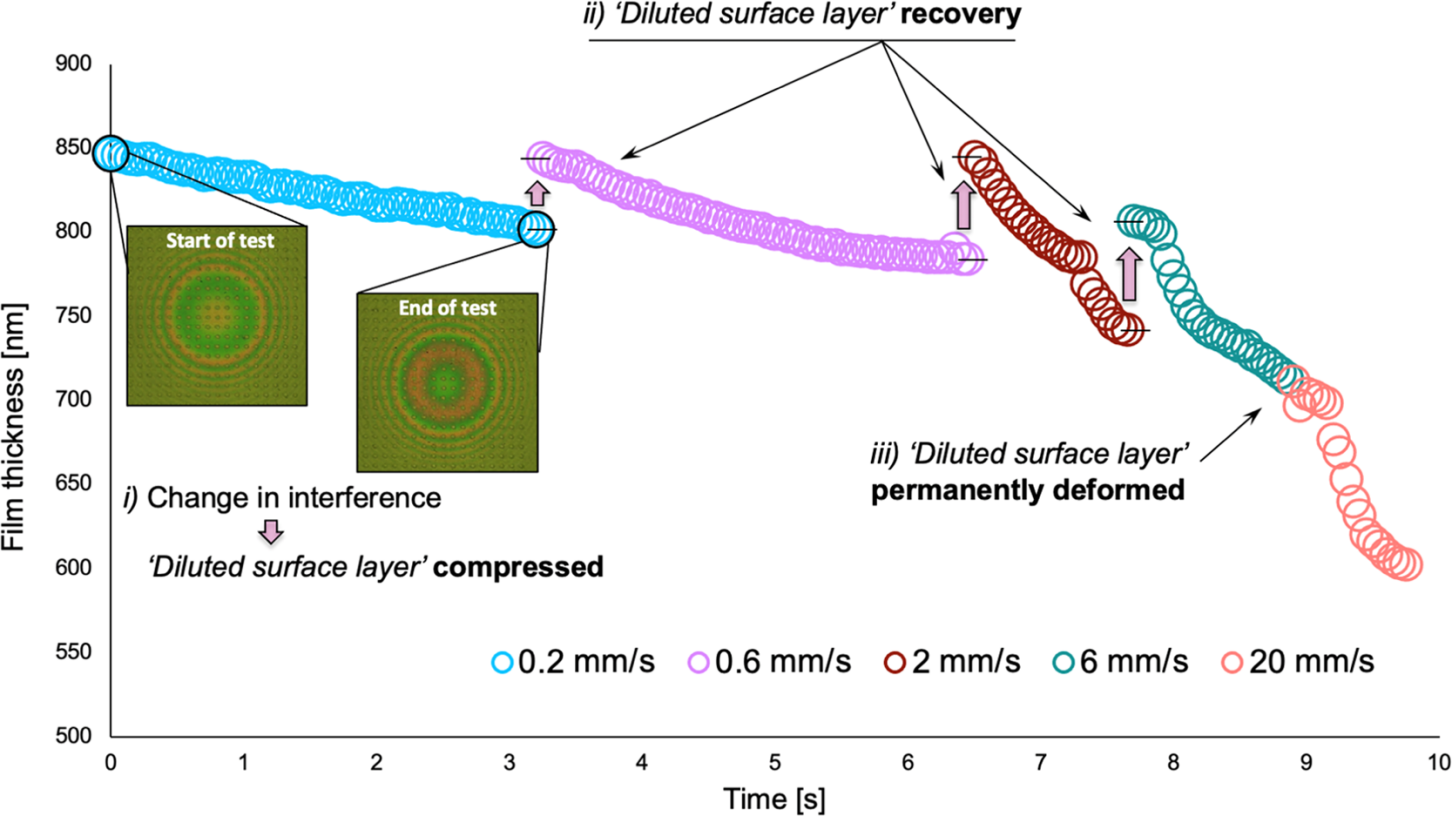
Combined CPB-IL film thickness captured for the Ø5 – *d*0.2 disc specimen (sliding speed: 0.2, 0.6, 2, 6, and 20 mm/s, applied load: 5 N; lubricant temperature: 25 °C). Inset: interferences fringes obtained initially and after 3 s.

The combined CPB-IL film thickness was measured for all tests on CPB-coated samples. Figure 7 shows one example for a test carried out with the Ø5 – *d*0.2-textured sample. As sliding speed increases, the CPBs are compressed. To avoid situations where the CPBs are removed completely, leading to direct contact between the disc’s chromium layer and the steel ball (as occurred in Figure 6), the test duration was adjusted accordingly for each sliding speed step. In each of these tests, the first 0.2 s represent the loading step. This initial, static period can be identified in the film thickness chart for the 6 mm/s speed case (Figure 7), where the recorded values show an accelerated collapse of the CPBs as soon as sliding motion starts.

Although not shown in Figure 7, the contact was unloaded after each speed measurement for a period of 60 s. This highlights the behavior of the CPB-“layered structure”, introduced in ref (34) and discussed above. It is likely that in this example, the “diluted surface layer” is approximately 50 nm, reduces in thickness during the first two speed measurements (0.2 and 0.6 mm/s), and more rapidly during the third speed measurements (2 mm/s). During this latter step, film reduction gradient changes (at ∼800 nm), probably indicating that the solid-like “bulk layer” is beginning to support the applied load. A similar behavior is observed at 6 mm/s. Figure 7 shows the almost complete recovery of the “diluted surface layer” between the first three speed steps (indicated by the arrows), followed by a partial recovery between the third (2 mm/s) and fourth (6 mm/s) speed steps. However, following the fourth step, no recovery occurs which suggests permanent deformation of this top layer occurred at a combined CPB-lubricant film thickness of ∼700 nm. At 20 mm/s, the film decays more rapidly due to increased sliding distance.

### 4.2 Parametric Summary of LST–CPB Friction

**Figure 8:**
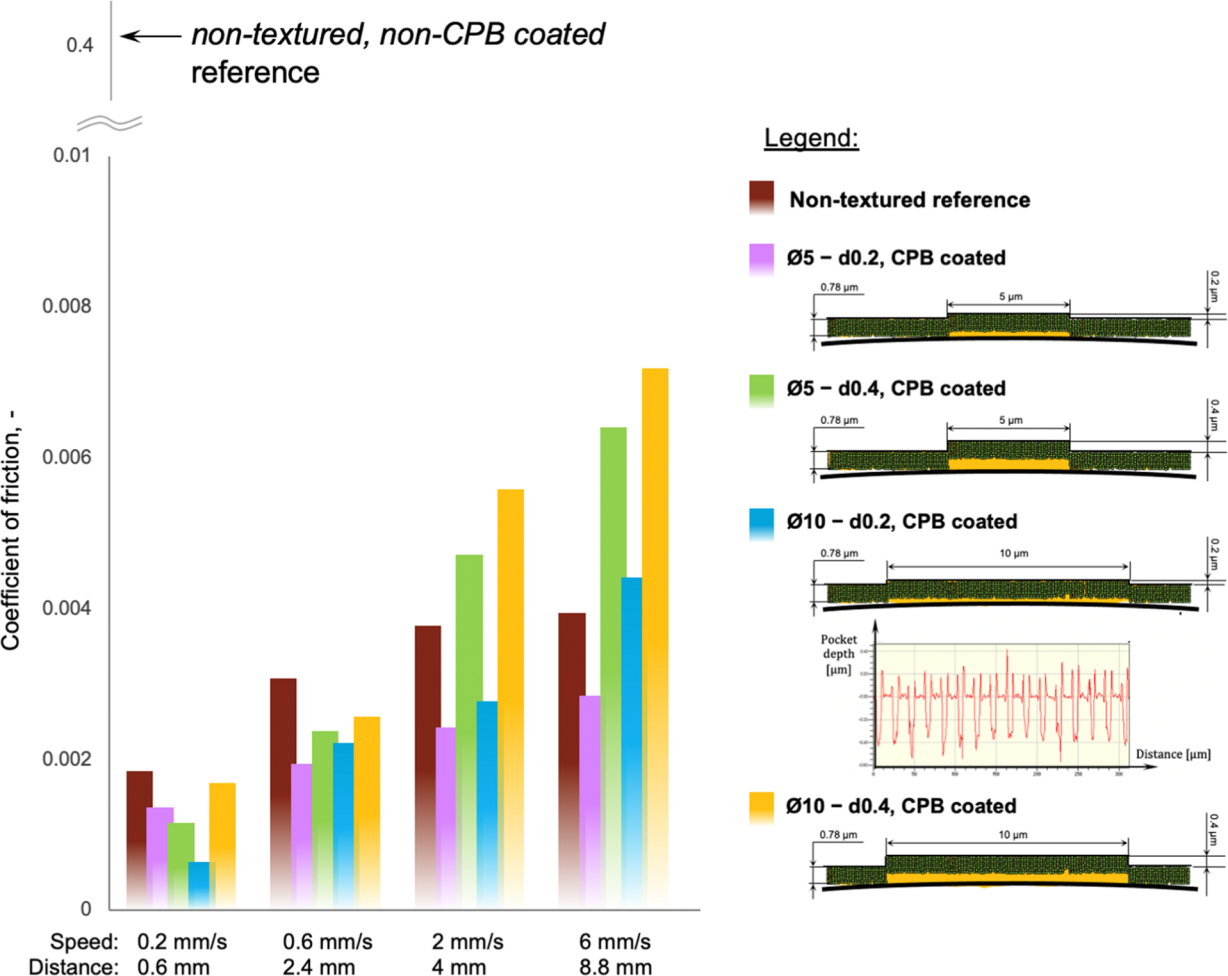
Friction comparison between all CPB-coated textured samples and the non-textured reference.

Figure 8 show friction versus sliding speed and distance performance of all four textured samples (with varying dimple depth and diameter) and the non-textured reference. For all specimens, friction force increases with sliding speed and distance due to decreasing film thickness. Initially, the non-textured specimen shows highest friction possibly due to its larger contact area. Then, as sliding progresses, the specimens with deepest pockets show highest friction probably due to increased squeeze flow of IL out of the contact leading to a thinner lubricant film. However, the shallower Ø5 *– d*0.2 pocketed consistently shows lower friction probably due to enhanced anchoring of the polymer brushes combined with minimum squeeze flow.

The observed improvements for textured specimens are attributed to the lateral support offered by the polymer brushes situated inside the textured features (i.e.*,* the “reinforced tough layer”) to the brushes grafted outside the pockets (i.e.*,* the “concentrated bulk layer”), thus delaying the decline in film thickness. Naturally, polymer brushes situated inside deeper pockets offer less support and lead to greater reduction of the CPBs on the sample’s surface. At the lowest sliding speed of 0.2 mm/s, the textured pattern Ø10 – *d*0.2 displayed the lowest frictional response, down to a coefficient of friction of just 0.0006. This improved performance of Ø10 – *d*0.2 is attributed to an increased material pileup around the edges of the pockets (i.e.*,* 200 nm tall spikes, generated by the laser texturing process—Figure 8, Legend). The laser parameters were deliberately modified to achieve these spike features in order to alleviate CPB wear compared to regular pockets. When polymer brushes were grafted onto this textured surface, the pileup spikes offered additional lateral support to the “concentrated bulk layer” and the “reinforced tough layer” of the CPB structure, reducing their compression and thus boosting their friction performance.

### 4.3 Impact of Abrasive Particles on the CPB Film

**Figure 9:**
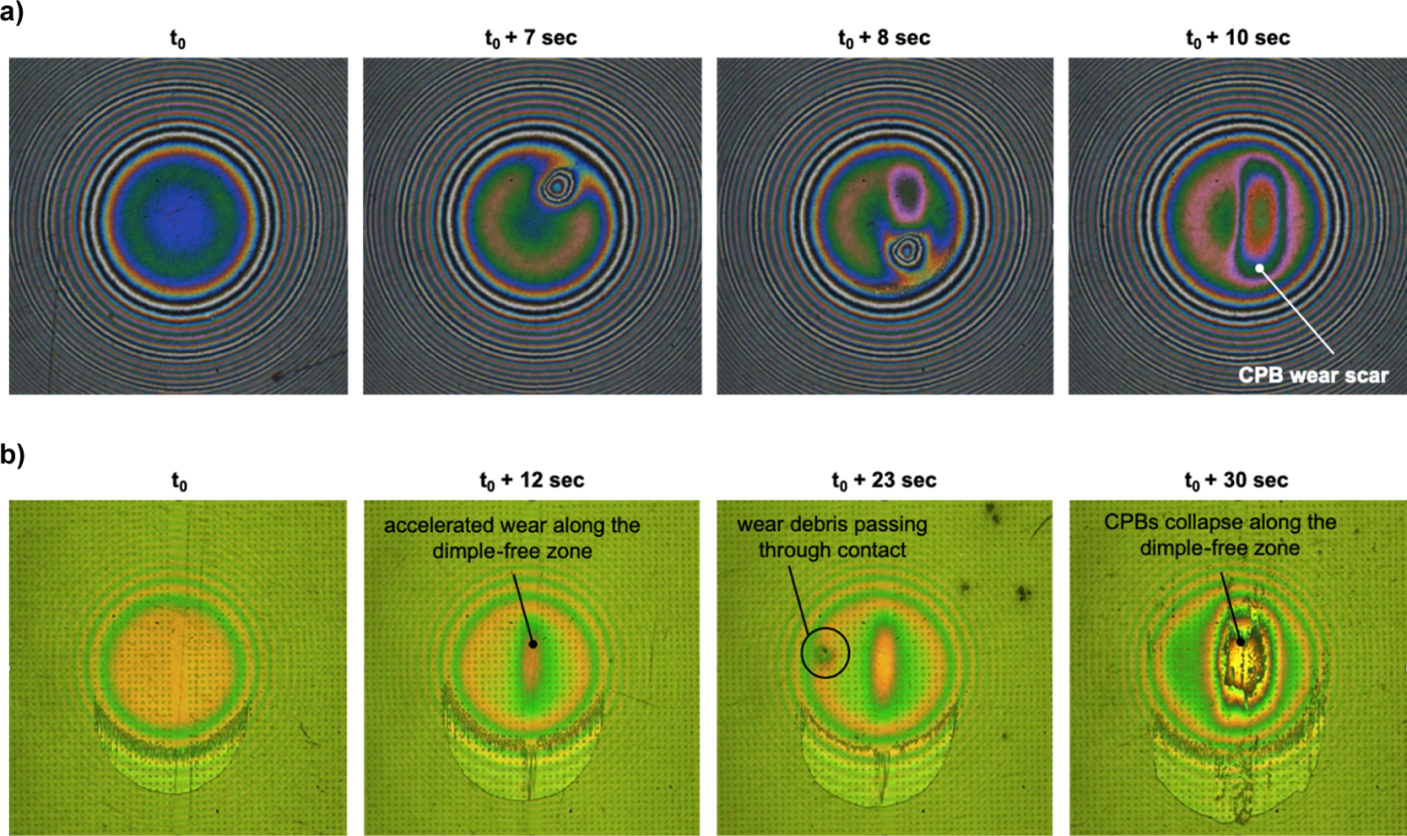
(a) Wear debris particle passing through a CPB-coated, non-textured contact and the subsequent wear damage of the CPB layer; (b) successive positions of a CPB-coated, laser-textured contact (Ø5 – *d*0.2) showing: accelerated wear along the dimple-free area, reduced damage due to wear debris passing through the textured area, and the collapse of the combined CPB-IL film.

The three chromatic LED images in Figure 9a (and Videos S5 in Supporting Information) show how a wear debris particle travels along the contact, damaging the CPB film through abrasion, leading to its collapse. Contrastingly, the halogen light image in Figure 9b shows a textured contact in which CPB wear initiates and gradually progresses along the dimple-free zone. However, beneficially, wear particles which enter the contact along the textured area do not cause any damage to the CPB coating. This may be due, as we have recently suggested,^34^ to surface texture providing an additional third layer inside the concave space of the pockets that increases wear resistance along the textured region by suppressing the propagation of CPB failure by offering additional lateral support to the surface and bulk layers.

Considering the CPB wear behavior illustrated in Videos S5 and S6, it is suggested that although CPB–LST combinations significantly reduce friction force (by up to 99.4%), this should currently only be considered in controlled wear environments free from abrasive conditions, such as static sealing, near-vacuum environments, or space tribology applications.

## 5 Conclusions

The ability of the grafted CPB to reduce friction and wear in sliding bearings is the focus of intense research. However, most studies to date were performed under mild or moderate test conditions, where polymer brushes were grafted onto the moving surface of a conformal contact (i.e.*,* low contact pressures). To understand the impact of CPBs, and their enhancement from LST, under severe contact conditions, we used a non-conformal contact subjected to sliding speeds up to 2000 mm/s and pressures of 25 MPa.

Measurements of friction, film thickness, and wear response (all obtained in situ and simultaneously) were performed using a high-speed, dual interferometry technique coupled to a custom-built, ball-on-disc test apparatus. Various textured patterns of pockets with different depths and diameters were compared against their corresponding non-textured, CPB-coated, and non-CPB coated references and yielded the following conclusions:

- Observed transient film thickness reduction at a constant sliding speed is caused by hydrodynamic squeeze flow of the IL as the countersurface approaches the compliant CPB surface. Here, the concomitant increase in the shear rate increases friction due to Newtonian viscous shear losses.
- At low sliding speeds (corresponding to short distance performance), the combined CPB–LST approach leads to average friction reductions of more than 99%.
- Pocketed surfaces are beneficial in terms of wear probably due to the lateral support that the polymer brushes situated inside the textured features (i.e.*,* the “reinforced tough layer”) offer to those grafted outside the pockets (i.e., the “concentrated bulk layer”)*.*
- Shallower pockets reduce friction to the most and offer increased lateral support for the polymer brushes grafted onto the disc surface and thus increase durability. This agrees with results obtained using one of the test samples with exceptional material pileup around the pocket edges, which were shown to provide additional, artificial lateral support and result in the most significant reduction in friction.
- At sliding speeds greater than 60 mm/s, CPB layers are removed instantly. This may be attributed to one or a combination of drivers (including sliding distance, shear stress, lifetime of the polymer brushes’ adhesion points, IL squeeze flow, and stress concentration-driven peeling), which completely removes the CPBs from the contact.
- Wear of CPB on textured surfaces initiates and gradually progresses along the pocket-free zone located in the center of the contact. However, in non-textured samples, abrasive wear particles act to immediately collapse the CPB layer; this suggests that pockets suppress the propagation of CPB failure through additional lateral support offered to the surface and bulk layers.
- Carefully selected surface texture can increase the durability of the CPBs layer by up to 34%, while simultaneously reducing the friction coefficient in extended sliding tests.

The practical implication of the current findings is that a combined CPB–LST approach could prove an excellent means of reducing frictional response (by up to 99.4%) in non-conformal bearings functioning under controlled abrasive wear conditions.

## Supporting Information Available

Preparation and characterization of the CPB (PDF)
Interferometry movie captured for one CPB-coated sample, starting from the moment the sliding movement began until the moment of CPB collapse (MOV)
Wear behavior of CPBs grafted onto the non-textured test sample (MOV)
Wear behavior of the CPBs grafted onto the Ø5 – *d*0.2 textured pattern (MOV)
Wear behavior of the CPBs grafted onto the Ø10 – *d*0.2 textured pattern (MOV)
Wear debris particle passing through a CPB-coated, non-textured contact and the subsequent wear scar showing the damage of the CPB layer (MOV)
Interferometry movie captured for one CPB-coated, laser-textured sample (Ø5 – *d*0.2) showing accelerated wear along the dimple-free area, reduced damage due to wear debris passing through the textured area, and the collapse of the combined CPB-IL film (MOV)

## Supplementary Material

am2c00725_si_001.pdf

am2c00725_si_002.mov

am2c00725_si_003.mov

am2c00725_si_004.mov

am2c00725_si_005.mov

am2c00725_si_006.mov

am2c00725_si_007.mov
