# Supplementary material for: Exploiting the Synergy between Concentrated Polymer Brushes and Laser Surface Texturing to Achieve Durable Superlubricity
Source: ACS Appl Mater Interfaces. 2022 Mar 25;14(13):15818–29. doi: 10.1021/acsami.2c00725 (PMC9007417; doi:10.1021/acsami.2c00725)
Supplement: Supplementary file 1 — am2c00725_si_001.pdf [file am2c00725_si_001.pdf]

Supporting Information

Exploiting synergies between concentrated polymer brushes and laser surface texturing to achieve durable superlubricity

Sorin-Cristian Vlădescu[a,c*], Chiharu Tadokoro[b], Mayu Miyazaki[c], Tom Reddyhoff[a], Takuo Nagamine[b], Ken Nakano[c], Shinya Sasaki[d], Yoshinobu Tsujii[e],

[a] Tribology Group, Department of Mechanical Engineering, Imperial College London, South Kensington, Exhibition Road, SW7 2AZ, London, United Kingdom

[b] Department of Mechanical Engineering, Saitama University, 255 Shimo-Okubo, Sakura, Saitama 338-8570, Japan

[c] Faculty of Environment and Information Sciences, Yokohama National University, 79-7 Tokiwadai, Hodogaya, Yokohama 240-8501, Japan

[d] Tokyo University of Science, Department of Mechanical Engineering, Tokyo, Japan

[e] Institute for Chemical Research, Kyoto University, Gokasho, Uji, Kyoto 611-0011, Japan

Corresponding author: s.vladescu12@imperial.ac.uk

The PDF file includes:

Legends for movies S1 to S6
Section S1
Figures S1 and S2

Other Supporting Information for this manuscript includes the following: Movies S1 to S6

Movie S1.

Interferometry movie captured for one CPB coated sample, starting from the moment the sliding movement begun, until the moment of CPB collapse.

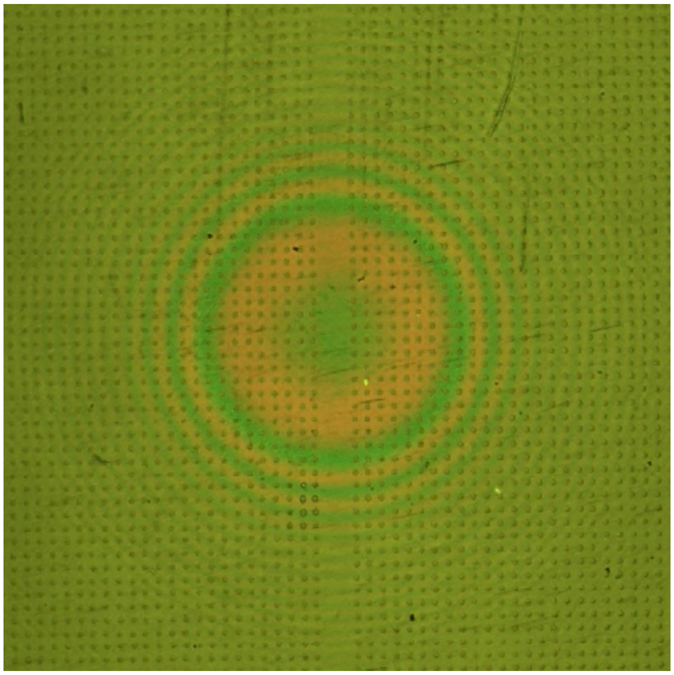

Movie S2.

Wear behaviour of the concentrated polymer brushes grafted on the non-textured test sample.

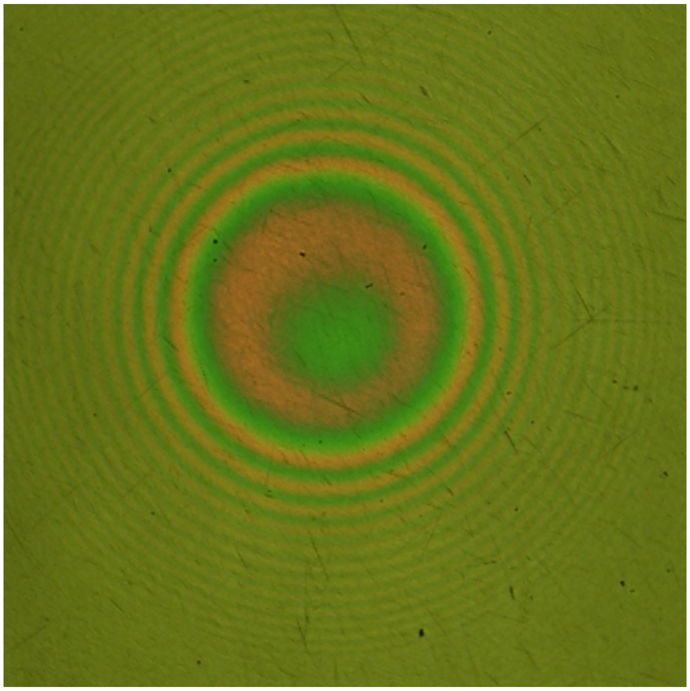

Movie S3.
Wear behaviour of the concentrated polymer brushes grafted on the *Ø5 − d0.2* textured pattern.

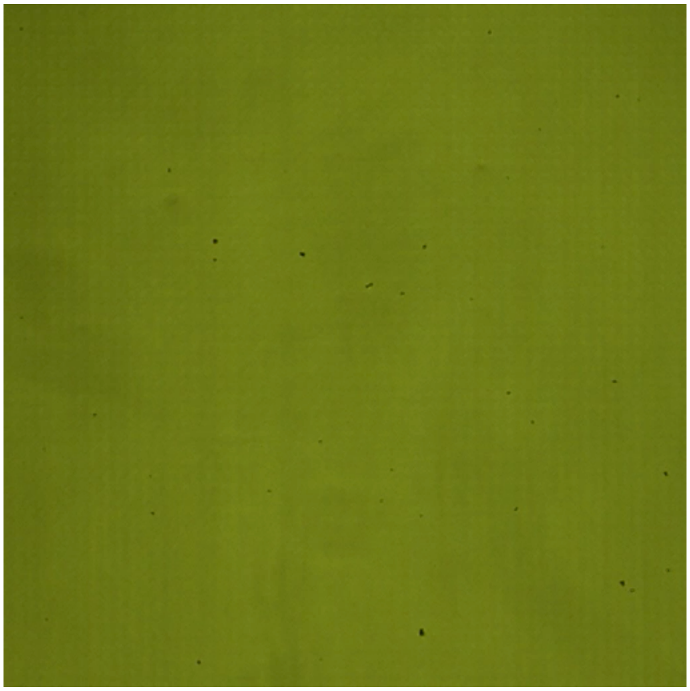

Movie S4.
Wear behaviour of the concentrated polymer brushes grafted on the *Ø10 − d0.2* textured pattern.

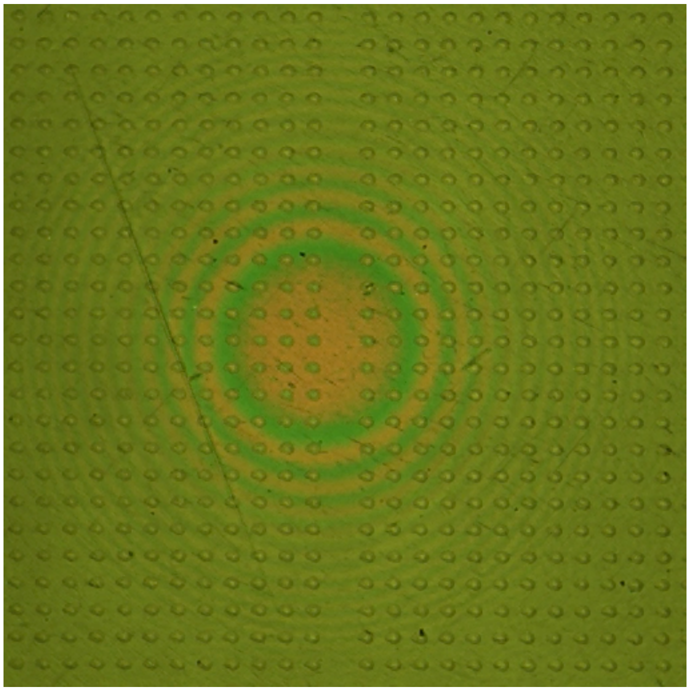

Movie S5.
Wear debris particle passing through a CPB coated, non-textured contact and the subsequent wear scar showing the damage of the CPB layer.

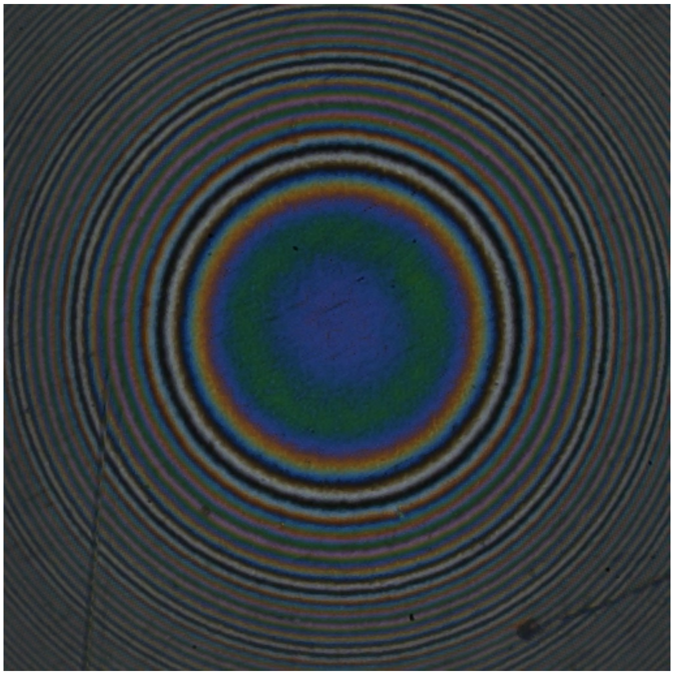

Movie S6.
Interferometry movie captured for one CPB coated, laser textured sample (Ø5 − d0.2) showing accelerated wear along the dimple-free area, reduced damage due to wear debris passing through the textured area, and the collapse of the combined CPB-ionic liquid film.

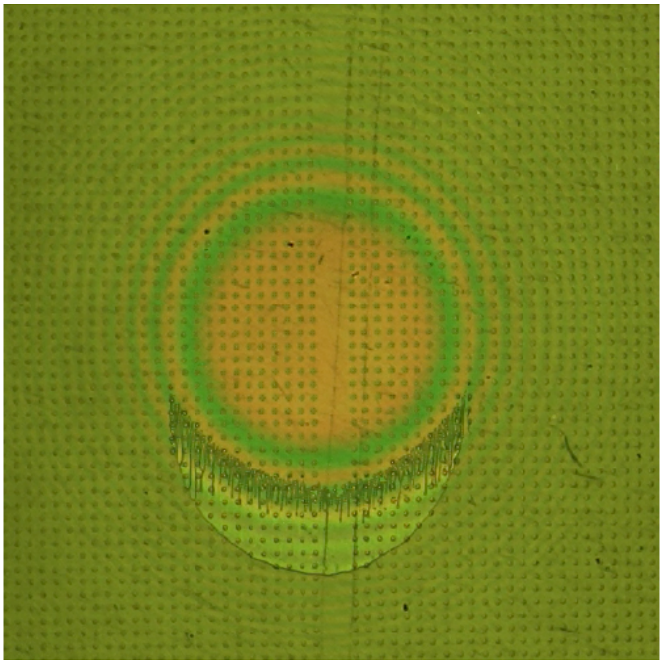

Section S1 – Preparation and characterization of concentrated polymer brushes

The chromium-coated fused silica discs were cleaned by ultrasonication in acetone, chloroform and 2-propanol followed by UV-ozone irradiation (PC440, Meiwafosis Co., Ltd., Tokyo, Japan), treated with an ethanol solution containing NH3 (0.24 M) and tetraethylsilaen (0.03 M) to fabricate a nanometre-thick silica layer, and finally cleaned by ultrasonication in ethanol. On the surface of the obtained discs, the polymerization-initiating group was immobilised by dipping them for 12 hours in an ethanol solution containing (2-bromo-2-methyl)propionyloxypropyl trimethoxysilane (1 wt%) and aqueous NH3 (10 wt%) while repeatedly cleaning the discs with ethanol. For half of the discs' surface area, the immobilized initiator was decomposed by UV-ozone irradiation for 10 minutes using a Si-wafer mask. The surface-initiated atom transfer radical polymerization was carried out at 60 ºC and 400 MPa for 2 hours in a deoxygenated solution of anisole (75 wt%) containing methyl methacrylate (2.3 M), ethyl 2-bromoisobutyrate (0.012 mM), Cu(I)Br (15 mM), Cu(II)Br2 (1.7 mM) and 4,4′-dinonyl-2,2′-bipyridine (33 mM). After the polymerization, the solution was analysed by gel permeation chromatography (Shodex GPC-101 high-speed liquid chromatography system) to determine the Mn and Đ values of free polymers as well as the monomer conversion. The PMMA-grafted discs were washed by ultrasonication in tetrahydrofuan to remove the physisorbed free polymers and impurities and analysed by ellipsometry to determine the thickness of PMMA layer in dry state. To estimate the σ and σ* values, the Mn and Đ values of the graft polymers were reasonably assumed to be equal to those of the free polymers, which was experimentally demonstrated[39].
